# Correction: Iron-catalyzed three-component 1,2-azidoalkylation of conjugated dienes *via* activation of aliphatic C–H bonds

**DOI:** 10.1039/d5sc90119g

**Published:** 2025-05-30

**Authors:** Zhen-Yao Dai, Chenxi Lin, Derek B. Hu, Jennifer M. Schomaker

**Affiliations:** a Department of Chemistry, University of Wisconsin–Madison Madison Wisconsin 53706 USA schomakerj@chem.wisc.edu

## Abstract

Correction for ‘Iron-catalyzed three-component 1,2-azidoalkylation of conjugated dienes *via* activation of aliphatic C–H bonds’ by Zhen-Yao Dai *et al.*, *Chem. Sci.*, 2025, **16**, 6336–6344, https://doi.org/10.1039/D5SC00307E.

The authors regret that in the original paper, the incorrect funding number was given in the Acknowledgements section to acknowledge the funding received by Jennifer M. Schomaker from the National Science Foundation. The Acknowledgements should read: J. M. S. is grateful to NSF (CHE-2247217).

The Royal Society of Chemistry apologises for these errors and any consequent inconvenience to authors and readers.

